# Evaluation of left ventricular diastolic function of patients with coronary heart disease by ultrasound images on bilateral filtering image noise reduction algorithm combined with electrocardiogram

**DOI:** 10.12669/pjms.37.6-WIT.4886

**Published:** 2021

**Authors:** Wen Li

**Affiliations:** 1 Wen Li, Master of Medicine. Electrocardiogram Room, Taizhou People’s Hospital, Taizhou, 225300, Jiangsu, China

**Keywords:** Bilateral filtering image noise reduction, Ultrasound images, Electrocardiogram, Coronary heart disease, Left ventricular diastolic function

## Abstract

**Objective:**

To explore the evaluation of left ventricular diastolic function (LVDF) in patients with coronary heart disease (CHD) using ultrasound images (UI) combined with electrocardiogram (ECG) on bilateral filtering image noise reduction algorithm (BFINRA).

**Methods:**

A BFINRA was constructed, and 60 subjects who were investigated were divided into a control group (CG) from June 2019 to November 2019 in Taizhou People’s Hospital, a myocardial infarction group (MIG), and an angina pectoris group (APG). The patient’s LVDF was examined by two-dimensional electrophoresis (2DE) and real-time three-dimensional echocardiography (RT-3DE) combined with ECG. The results showed BFINRA could improve UI quality.

**Results:**

Clinical data indicated there were no substantial differences in age, gender, and fasting blood glucose of all subjects. 2DE examination results showed the left ventricular end-diastolic volume (LVEDV), left ventricular end-systolic volume (LVESV), and early diastolic mitral blood flow velocity / early diastolic mitral annulus velocity (E/E’) of MIG were much higher than CG (P<0.05), while the left ventricular ejection fraction (LVEF), E / late diastolic mitral blood flow velocity (E/A) and E’ peak value were sharply decreased (P<0.05);LVESV and E/E’ of APG were increased dramatically (P<0.05), while E peak, E/A and E’ peak were decreased greatly. RT-3DE examination results indicated LVEDV and LVESV of MIG were considerably higher than CG (P<0.05), while LVEF and macrophage resistance factor (MRF) were enormously decreased (P<0.05);LVEDV and LVESV of APG were greatly increased (P<0.05). However, LVEF and MRF were not changed significantly (P>0.05). LVEDV had a remarkable difference (P<0.05), but LVESV and LVEF had no obvious differences (P>0.05). The electrocardiogram results illustrated the increase in QT dispersion (QTd) of MIG and APG was statistically significant (P<0.05) compared with CG, while the negative increase of P-wave terminal force in lead V1 (PTFV1) also had a statistical significance (P<0.05). Correlation analysis revealed that MRF and PTFV1 had positive correlation, while MRF and QTd showed a negative correlation.

**Conclusion:**

The combination of UI and ECG could better assess LVDF in CHD patients.

## INTRODUCTION

RT-3DE has greatly improved the application of ultrasound diagnosis in the evaluation of cardiac morphology and function, which does not rely on geometric assumptions and can reflect the dynamic changes of the heart chamber volume in real-time to obtain the left ventricular volume-time curve (VTC).^1^-^3^ Thus, RT-3DE provides a non-invasive, simple, and new approach to evaluate heart function. The bilateral filtering can optimize echocardiography, providing more accurate information. There are more speckle noises in ultrasound images, so that the effectiveness of doctors’ diagnosis of diseases and evaluation of curative effects are affected.^4^-^6^ At present, many algorithms have been applied to the denoising of medical ultrasound images, such as wavelet transform, non-average denoising, and bilateral filtering denoising.^7^,^8^ Among them, bilateral filtering can optimize the echocardiography and provide more accurate information.

To evaluate the differences in the diagnosis of patients with coronary heart disease by electrocardiogram, two-dimensional echocardiography, and three-dimensional echocardiography, the optimized bilateral filtering algorithm was adopted to denoise the echocardiogram, and then the UI was combined with ECG to evaluate the LVDF of CHD patients. The study was intended to provide a reference for improving the diagnosis efficiency of coronary heart disease and the treatment effect of patients.

## METHODS

Gaussian filtering was to assign different spatial position weights to pixels in the image range and obtain the pixel values of the points to be processed by the weighted average method, as shown below.


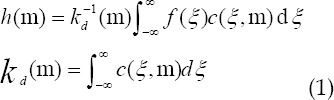


Here, *h(m)* represented the output value of the pixel, *f(ξ)* and c(*ξ*,m) represented the set of input pixels and the spatial position weight, respectively;and *Kd*(m) represented the unitization factor to ensure that the absolute smooth position gray value remained unchanged.

It showed from the above that Gaussian filtering only considered the spatial position relationship among pixels, but ignored the processing of image edge information. Edge information expressed abstract information in the images, such as texture detail information and locations with greater color contrast.^9^ Bilateral filtering solved the edge information retention through pixel similarity weights. They were expressed in the following.


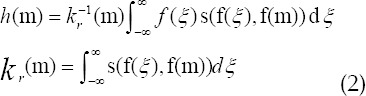


Where, s(f(*ξ*),f(m)) and *k_r_*(m) stood for the pixel similarity weight and unitization factor, respectively. A new bilateral filter function was obtained through the combination of the pixel similarity weight and unitization factor, which not only considered the spatial position relationship, but also the degree of pixel similarity. The theoretical equations were expressed as follows.


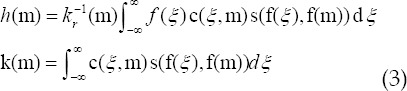


The integral form of equation (3) was discretized and rewritten, and the calculation of bilateral filtering limited in the neighborhood was expressed in the following.


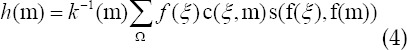


Where Ω represented the set of neighborhood pixels around the target points, and the basis for the realization of bilateral filtering was formed by equation (4).Forty patients with CHD were selected for the experimental group.

### Inclusion criteria

The patients were diagnosed with CHD, had typical changes in biochemical markers of myocardial necrosis, had no hypertension, diabetes, and other cardiovascular, and metabolic diseases, had no sick sinus syndrome, atrial fibrillation, and pre-excitation syndrome, and agreed to this experiment and signed informed consent forms.

### Exclusion criteria

Patients had a severe mental illness, severe liver and kidney diseases, severe blood disease, and other severe types of heart diseases.

Twenty healthy people were selected from recruitment as the CG. The study was approved by the Ethics Committee of the Hospital (dated March 16, 2021).

The Philips Ie33 color Doppler ultrasound system was for 2DE and RT-3DE examinations. The ultrasound system was equipped with an S5-1 probe, X3-1 matrix 3D probe, and QLAB8.0 image analysis software. The patient was required to rest peacefully for about 10 minutes, and then, lie down on the left side while the chest lead ECG is being recorded. First, the S5-1 probe can routinely examine echocardiographic on the subjects. In the direction of the long axis of the left ventricle, the LVESD and left ventricular end-diastolic diameter (LVEDD) were measured. The four-chamber and two-chamber views were obtained from the cardiac apex to make the endocardium in each view be displayed. Besides, the double-plane Simpson’S method can measure LVEDV and LVESV and calculate LVEF. The above parameters applied the average of three consecutive cardiac cycles.

The QLAB8.0 image analysis software can open the images to select the mitral annulus level of the four-chamber and the two-chamber heart views at the apex. The software can measure the heart rate (HR), LVEDV, end-systolic volume (ESV), and ejection fraction (EF);and the ratio of early and late diastolic volume was calculated from end-systole to mitral valve opening image. The sequence analysis was started to automatically obtain the VTC through the software, and the slope of each point on the curve was calculated based on the volume data at each time point of the VTC to obtain the average filling rate (MFR).

The standard synchronous 12-lead electrocardiogram was applied to detect all the subjects, and the amplitude and paper speed was set to 10mm/mv and 25mm/s, respectively. The record of each lead was more than six cardiac cycles. Based on sinus rhythm, the vector of PTFV1 referred to the value obtained by multiplying the amplitude of the biphasic P-wave negative phase wave in lead V1 and time, and its unit was mm•s. The measurement of QTd was related to the measurement of QT interval, so the number equaled maximum QT less the minimum QT on the different leads of the synchronized 12-lead ECG. There were synchronization and asynchrony in each lead of the ECG, and the synchronized 12-lead ECG was to observe the waveform of the same cardiac cycle to reduce the influence of heart rate variability on the measured value of the QT interval and to decrease errors.

### Statistical analysis

The t-test can compare the means between the two groups, while the one-way analysis of variance was applied to the comparison of the means among multiple groups. Besides, the LSD method was used for pairwise comparison. The linear correlation analysis of binary variables was analyzed with the Pearson method, and the correlation was expressed with the Pearson correlation coefficient. There was a difference with statistical meaning at *P*<0.05.

## RESULTS

**Fig.1:**
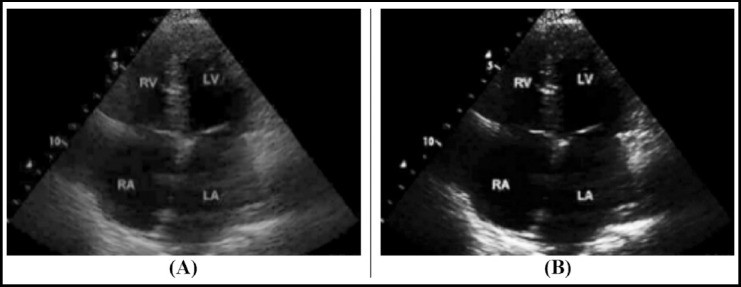
Comparison of echocardiography before and after optimization. Note: (A) was the image before optimization, and (B) was the image after optimization.

As shown in Fig.1, Fig.1(B) had a clearer layout and more obvious structure after bilateral filtering noise reduction, beneficial for observation and diagnosis.

Based on the diagnosis results, all subjects were divided into 3 groups: the CG (20 subjects), MIG (19 subjects), and APG (21 subjects). The parameters, including gender ratio, age, and HR of the patients in each group were shown in Table-I. There was no significant meaning among the subjects in each parameter (*P*>0.05).

**Table-I:** Summary of subjects’ clinical data

	*CG*	*MIG*	*APG*
Gender (male / female)	10/10	10/9	11/10
Age (year)	55.3±9.8	55.9±10.4	53.6±8.5
HR (bmp)	67.4±6.7	63.5±7.2	68.2±9.8
Systolic pressure (mmHg)	126.5±5.7	128.2±7.8	130.7±7.4
Diastolic pressure (mmHg)	76.4±6.8	79.4±6.9	78.3±7.2
Fasting blood-glucose (mM)	5.08±0.76	5.15±0.48	5.17±0.55
Total cholesterol (mM)	4.65±1.23	4.16±1.62	4.78±1.43
Triglyceride (mM)	1.18±0.34	1.17±0.16	1.21±0.32

**Fig.2:**
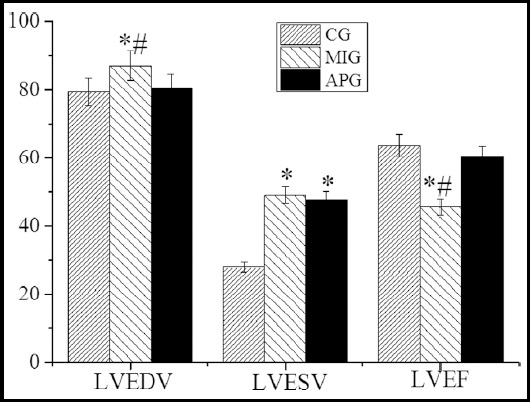
Parameters related to LVDF measured by 2DE. ***Note:*** *expressed P<0.05 when MIG and APG were compared with CG; and # expressed P<0.05 when MIG was compared with APG.

**Fig.3:**
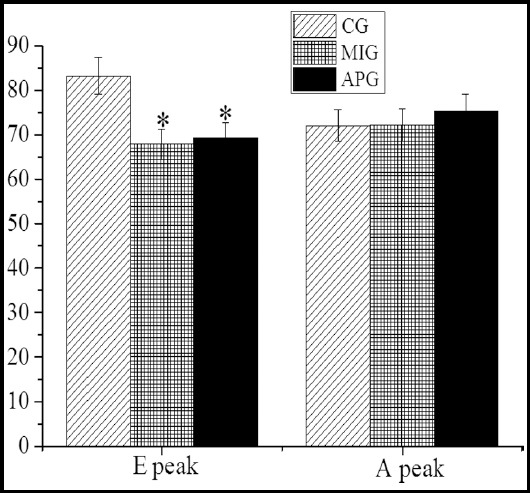
Parameters related to LVDF measured by 2DE. ***Note:*** E stood for early diastolic mitral blood flow velocity;A stood for late diastolic mitral blood flow velocity, and E’ expressed early diastolic mitral annulus velocity. * expressed P<0.05 when MIG and APG were compared with CG; and # expressed P<0.05 when MIG was compared with APG.

**Fig.4:**
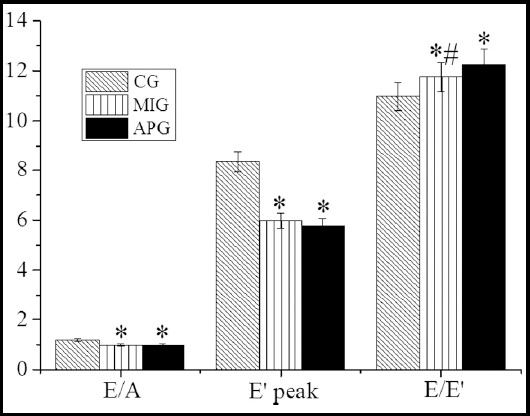
Parameters related to LVDF measured by 2DE. * indicated P<0.05 compared MIG and APG with CG; and # indicated P<0.05 compared MIG with APG.

In Fig.2, 3, and 4, the measurement results of LVDF were illustrated by the 2D echocardiography. It showed that the LVEDV, LVESV, and E/E’ of the patients in MIG were enormously improved (*P*<0.05) compared with the CG, while LVEF, E peak, E/A, and E’ peak of the patients in MIG were decreased steeply (*P*<0.05). The LVESV and E/E’ of the patients in APG were increased hugely (*P*<0.05), while E peak, E/A, and E’ peak the patients in APG were decreased dramatically (*P*<0.05). Therefore, there were substantial differences in LVEDV, LVEF, and E/E’ by comparing MIG with APG (P<0.05).

**Fig.5:**
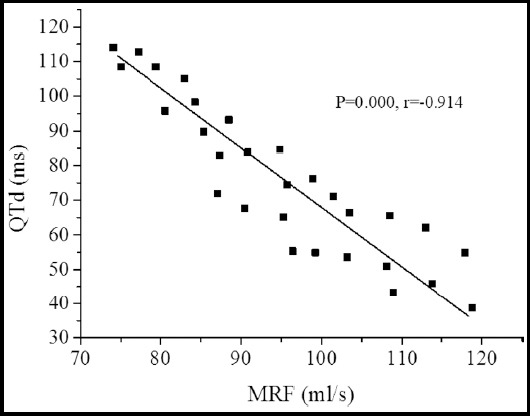
Correlation analysis of MRF and QTd.

LVEDV and LVESV of the patients in MIG were greatly increased (*P*<0.05) compared with CG, while LVEF and MRF were hugely decreased (*P*<0.05). LVEDV and LVESV of the patients in APG were also enormously increased (*P*<0.05) compared to CG, while LVEF and MRF were not changed obviously (*P*>0.05). Compared MIG with APG, there was a substantial meaning between LVEF and MRF (*P*<0.05).The LVEDV results measured by the two methods were greatly different (*P*<0.05), while there were no obvious differences in the results of LVESV and LVEF measured by the two methods (*P*>0.05).There was a positive correlation between MRF and PTFV1 (*P*=0.000, and *r*=0.895). However, MRF was negatively correlated with QTd (*P*=0.000, and *r*=-0.912) in Fig.5.

## DISCUSSION

There is a lot of speckle noise in ultrasound images, which greatly affects the accuracy and effectiveness of ultrasound imaging, reduces the quality of the image, and limits the development of automatic diagnosis technology for ultrasound images.^9^ In recent years, the methods used for ultrasonic image denoising can be classified into adaptive denoising, anisotropic diffusion denoising, non-local mean denoising, multi-scale denoising, and hybrid denoising.^10^,^11^ However, the denoising algorithm using only wavelet transform is not effective in removing speckle noise in medical ultrasound images.^12^ For bilateral filters, it has a strong denoising effect while also maintaining image edge details when dealing with image noise.^13^ In this study, the improved bilateral filtering algorithm was used to denoise the ultrasound image. The results suggested that the layout of the ultrasound image after bilateral filtering and denoising was clearer and the structure was more obvious, which was conducive to the observation and diagnosis of the patient’s condition.

Secondly, the difference between the results of quantitative assessment of cardiac function of patients with different methods were analyzed and compared. The results revealed that the LVEDV results measured by the 2DE and 3DE methods were quite different. The negative value of PTFV1 is due to the weakening or obstruction of left ventricular diastolic function in patients with CHD, which leads to a decrease in left ventricular compliance, resulting in a continuous increase in left atrial pressure in patients.^14^ QTd can reflect the instability of the ventricular myoelectricity and the heterogeneity of repolarization of the patinet, and the maximum value of QTd often appears in the ischemic lesion area or infarct area.^15^-^17^ The results of this study indicated that there was an obvious difference between the negative PTFV1 and QTd of patients with myocardial infarction and angina. Among them, the negative PTFV1 and QTd of patients with myocardial infarction were observably greater than those of patients with angina, which was basically consistent with the results of Karadeniz et al.^18^ The correlation between 3DE and ECG parameters was tested, and the results showed that there was a positive correlation between MRF and PTFV1, and there was a negative correlation between MRF and QTd. It is suggested that MRF, PTFV1 negative values, and QTd could be applied in the diagnosis of patients with CHD and in the identification of patients with angina pectoris and myocardial infarction.^19^-^20^

## CONCLUSION

The results demonstrated that 2DE and RT-3DE combined with ECG could effectively examine the changes in LVDF of CHD, and could be applied in clinic. This study provides a simple and easy operatiing process for the detection of LVDF in CHD, which can improve the efficiency of doctors and simplify the patient’s medical process. Due to the limitations of the experimental scale and scope, it was impossible to make effective recommendations for the clinical treatment of CHD. Therefore hopefully this aspect can be covered in future research.
